# Current SARS-CoV-2 Protective Strategies for Healthcare Professionals

**DOI:** 10.3390/biomedicines11030808

**Published:** 2023-03-07

**Authors:** Miriam Ting, John A. Molinari, Jon B. Suzuki

**Affiliations:** 1 Department of Periodontics, University of Pennsylvania, Philadelphia, PA 19104, USA; 2 School of Dentistry, University of Detroit Mercy, Detroit, MI 48221, USA; 3 Department of Graduate Periodontics, University of Maryland, Baltimore, MD 20742, USA; 4 Department of Graduate Prosthodontics, University of Washington, Seattle, WA 98195, USA; 5 Department of Graduate Periodontics, Nova Southeastern University, Fort Lauderdale, FL 33314, USA; 6 Department of Microbiology and Immunology (Medicine), Temple University, Philadelphia, PA 19140, USA; 7 Department of Periodontology and Oral Implantology (Dentistry), Temple University, Philadelphia, PA 19140, USA

**Keywords:** COVID, pathophysiology, prevention, SARS-CoV, mouthwash, disinfection, aerosol, sanitization, PPE

## Abstract

Severe acute respiratory syndrome coronavirus-2 (SARS-CoV-2) is responsible for the Coronavirus disease 2019 (COVID-19). COVID-19 was first reported in China in December 2019. SARS-CoV-2 is highly contagious and spread primarily via an airborne route. Hand hygiene, surgical masks, vaccinations and boosters, air filtration, environmental sanitization, instrument sterilization, mouth rinses, and social distancing are essential infection control measures against the transmission of SARS-CoV-2. This paper aims to provide healthcare professionals with evidence-based protective strategies.

## 1. Introduction

Starting in December 2019, a large number of people were hospitalized with severe pneumonia in Wuhan, China [1]. The etiologic agent was unlike any previously known pathogen. In January 2020, pharyngeal and respiratory swabs from these hospitalized patients revealed a new coronavirus, SARS-CoV-2. By March 2020, there were 98,192 confirmed cases of COVID-19, with a mortality rate of 2% [2]. On 11 March 2020, the World Health Organization (WHO) declared it a pandemic [3].

The routes for disease transmission from an infected SARS-CoV-2 person include contact with infected body fluids, contaminated surfaces or instruments, and airborne infectious particles [4]. Demonstration of an airborne route of viral transmission also had a significant impact on infection control in healthcare settings by emphasizing preventive practices against microbial aerosols. The potential for airborne microbial infection was already recognized before the discovery of specific infectious agents such as bacteria and viruses. Historical accounts of pneumonic plague reported the spread to non-infected patients who were not in direct contact. Apparently, the inhalation of the bacteria *Yersinia pestis* resulted in universally fatal bacterial infection [5]. In 1996, a publication reported that aerosolized *Mycobacterium tuberculosis* from a patient with active tuberculosis (TB) entered an airplane’s ventilation system and infected other passengers [6]. Measles virus, which is one of the most infectious respiratory pathogens, is also spread by virus-laden aerosols, has also been reported to spread infection to susceptible children through the ventilation system [7].

The oral cavity is one of the portals for SARS-CoV-2 entry and viral transmission. The health of the oral cavity may influence the transmissibility of the virus. Good oral health and meticulous oral hygiene may reduce the risk of SARS-CoV-2 infection or transmission [8,9]. 

## 2. Transmission of SARS-CoV-2

At the peak of the COVID-19 pandemic, many medical and dental clinics were forced to reduce patient access by limiting clinical activity to urgent care only. Overcrowding, an insufficient availability of respirators, inconsistent airborne infection control, and an inability to constantly sterilize contaminated environments were shown to contribute to infection caused by coronaviruses responsible for Severe Acute Respiratory Syndrome (SARS) and Middle East Respiratory Syndrome (MERS) [10,11,12,13,14]. Person-to-person cross-infection can occur through contact with infected COVID-19 patients via respiratory droplets from coughing or sneezing [15]. In addition, inhalation of infectious micro-droplets through the nose, mouth, as well as contact with SARS-CoV-2 through eyes could also result in the onset of COVID-19 disease [16]. 

Droplets or splatter are airborne particles of diameter greater than 50 μm. These particles eject forcefully from the operative area but are too heavy to be suspended in the air for an extended time and are only airborne briefly [17]. In contrast, aerosols are micro-droplet particles with diameters far less than 50 μm. These particles are small enough to stay airborne for an extended periods before contaminating environmental surfaces or being inhaled. Particles less than 5 μm are reported to be suspended in closed environment for longer periods of time [18]. For particles between 5–10 μm in size the suspension remains variable and is contingent upon room temperature, airflow, transient personnel, etc. [19]. Particles greater than 10 μm more quickly settle onto the floor or inanimate surfaces [19]. Smaller aerosol particles of diameter 0.5–10 μm can penetrate further in the respiratory tract and have the greatest potential for viral transmission [20,21,22]. 

The composition of an aerosol depends on the patient and procedures performed. It is mostly comprised of saliva, nasal and pharyngeal secretions, blood, bacteria and viruses suspended in these secretions [20]. 

The inability for physical distancing of clinicians and patients in an overcrowded settings have been demonstrated to result in higher rates of COVID-19 infection in hemodialysis rooms [23]. This information may be applied to other clinical settings with reference to physical distancing.

In the medical and dental facilities, high concentrations of aerosolized SARS-CoV-2 can increase occupational airborne infection risks. In the hospital, aerosol-generating procedures (AGP) include intubation, bronchoscopy, cardiopulmonary resuscitation or use of the nebulizer [24,25,26]. Table 1 summarizes medical procedures generating aerosols.

Increased aerosols were detected before and after intubation [27]. There were higher rates of infection in healthcare providers involved in intubation compared to controls [28]. The risk of infected aerosols exposure was highest among healthcare providers during the peri-intubation period [28]. Other data suggest a low rate of SARS-CoV-2 infection in anesthetists and intensivists performing endotracheal intubation [29]. More effective personal protective equipment during intubation may influence the risk during intubation. 

Extubation may present more opportunities for aerosolization. Extubation may trigger forceful coughing. The removal of the endotracheal tube during high air flows may cause aerosolization of the secretions in the tube.

During the SARS epidemic, tracheostomy insertion showed an increased transmission risk to healthcare providers compared to controls [30]. The process often involved bronchoscopy guidance which could create a leak from the ventilation circuit distal to the microbial filters. This would most probably cause aerosol generation. Disconnecting the endotracheal tube from the tracheostomy may also generate aerosol. Tracheostomy removal or change usually involves suctioning, which will cause forceful coughing. This may generate aerosolized particles from upper airways and the tracheostomy site.

Data from air sampling studies on aerosolization during bronchoscopy were contradictory [31]. One study reported that bronchoscopy without nebulized medication did not generate more aerosols [32]. But another bronchoscopy study focusing on influenza Ribonucleic acid (RNA) detected increased RNA-containing aerosols [27]. Thus, treating bronchoscopy as an aerosol-generating procedure remains a precaution. 

During use of high flow nasal oxygen devices, mouth opening allows flow to be diverted out of the oral cavity, generating aerosol from the nasal passages and upper airways [33]. The use of surgical face masks in conjunction with high-flow nasal oxygen can significantly reduce the dispersion of bioaerosols and may be a risk reduction strategy [33]. 

The suctioning of intubated patients and the disconnection of the ventilator is a source of aerosolization. This risk from airway suctioning is associated with healthcare worker infection rates during the SARS epidemic [31]. 

Nebulization can increase aerosol particle detection. These particles were generated from the nebulizer and not the patient. They should not, therefore, pose an infection risk. However, as a precautionary measure, sputum induction with nebulized saline is managed as an aerosol-producing procedure. 

Prolonged and forceful coughing may generate aerosols. Nasogastric tube insertion [34] and swallow assessment [35] often induces forceful coughing and should be considered aerosol-generating procedures. 

Dental aerosols may also include tooth shards and any materials used in the dental procedure such as abrasives used for air polishing [20,36]. Aerosols generated during dental procedures may remain suspended in the air for several hours and can spread up to 3 m from the source. Dental equipment-generated aerosolized particles may present as aerosol, droplets or splatter (0.001 to 50 µm) [37]. 

In the dental office, AGP include use of high-speed handpieces and other rotary instruments, ultrasonic instruments with water cooling systems, and air-water spray syringes [38]. Visible and non-visible droplets of water, blood, saliva, bacteria and viruses are created by these instruments [39]. Dental professionals are particularly at risk due to the inability to maintain adequate interpersonal distance during dental procedures that contact blood and saliva. Dental procedures have been shown to produce microbial-laden aerosols [39]. Table 2 summarizes dental devices and procedures that produce aerosols.

Coronaviruses have been detected in sputum, nasopharyngeal secretions, bronchoalveolar lavages, urine, tears, feces, conjunctival secretions, blood and lung tissues [40,41,42,43]. These coronaviruses can survive in sputum, serum, and feces for at least 96 h, in urine for 72 h [44], on surfaces for up to 9 days [45]. As mentioned above, SARS-CoV-2 can also survive outside the living cell, in droplets, aerosols and on surfaces; viability in aerosols can last for up to 3 h and can survive on stainless steel and plastics for up to 72 h [46].

Measles, SARS-CoV-2, and other airborne viruses in aerosol can be difficult to contain in clinical environments. Therefore, infection control strategies need to be adequately broad to encompass all transmission modes. SARS-CoV-2 is more contagious in symptomatic patients but can spread from human to human even in asymptomatic patients or patients with mild symptoms [47].

## 3. Pre-Appointment Screening

Telephone surveys of patients before their medical or dental appointments could aid in recognizing infected COVID-19 patients before they arrive at the dental practice. Telephone survey questionnaires may include body temperature, COVID-19 symptoms, contact with COVID-19 infected persons, and vaccination status. Patients with body temperatures greater than 37.5 degrees Celsius should proceed with caution.

## 4. Pre-Appointment Mouth Rinsing

Studies conducted during the early months of the pandemic demonstrated that SARS-CoV-2 was consistently detected in the saliva of 92% of COVID-19 positive patients [48]. As a result, the pandemic sparked research as to the antiviral properties of multiple mouthwash formulations [49], and the use of a pre-procedural mouth rinse has been suggested [50]. Previously, antimicrobial mouthwashes have been traditionally used to reduce oral bacteria, with a preprocedural mouthwash reported to be most effective in reducing the bacterial load in oral aerosols [51,52]. 

Historically, pre-procedural rinses were reported to be effective against several infectious viruses, including human immunodeficiency virus (HIV), herpes simplex virus (HSV), and hepatitis B virus (HBV) [50]. Commercial mouthwashes include active ingredients such as chlorhexidine gluconate (CHX), cetylpyridinium chloride (CPC), povidone iodine or polyvinylpyrrolidone iodine (PVP-I), hydrogen peroxide (H_2_O_2_), stabilized hypochlorous acid, dipotassium oxalate, zinc fluoride, sodium fluoride, eucalyptol, thymol, and menthol [53]. 

With reference to the strong in vitro sensitivity of SARS-CoV-2 to oxidizing agents, some studies suggested the use of 1% H_2_O_2_ for 30 s to reduce salivary viruses in vivo [54,55]. On the contrary, in vitro studies on H_2_O_2_, CPC, CHX, and PVP-I concluded that PVP-I at 0.5–1.5% or CPC should be preferred over H_2_O_2_ or CHX [49]. Some studies reported that mouthwashes containing CHX were not effective against SARS-CoV-2 [54,55]. However, the interaction of the mouthwash with oral environment and the rinsing effect of the mouthwash in the oral cavity may have differing in vivo effects compared to in-vitro. 

In a clinical study of non-hospitalized patients with asymptomatic or mildly symptomatic SARS-CoV-2 infection, 0.07% CPC mouthwash compared to placebo was associated with a significant increase in lysed SARS-CoV-2 [56]. Another clinical study reported that both CPC plus zinc mouthwash and CHX mouthwash significantly reduced SARS-CoV-2 viral load for up to 60 min after rinsing, and H_2_O_2_ significantly reduced SARS-CoV-2 for up to 30 min [57]. Clinically, all four mouthwashes (saline, 1% H_2_O_2_, 0.12% CHX, or 0.5% PVP-I) decreased viral load by 61–89% at 15 min and by 70–97% at 45 min [58]. The antiviral activity of mouthwashes may depend on the sensitivity of the SARS-CoV-2 lipid envelop to surfactant properties of the mouthwash disrupting the viral membrane. The surfactant disruption of the viral membrane is virucidal despite viral mutations [59].

## 5. Hand Hygiene

One component singled out for special mention in the “clean it first” category is hand hygiene. All infection control recommendations and guidelines stress the importance and clinical impact of this practice. Hand hygiene is the most important procedure for minimizing the potential for development of nosocomial infections [60,61,62,63,64,65]. Its primary purpose is the mechanical removal of transient microorganisms from the skin, preventing cross-contamination and cross-infection from contaminated hands.

The most frequently used classes of antimicrobial antiseptics currently available are CHX, parachlorometaxylenol, and triclosan. Each is capable of providing substantivity, a residual antimicrobial effect, after each succeeding handwash procedure during the day. The CDC is now recommending alcohol-based hand products (i.e., preparations containing 60% to 95% alcohol) as an option for routine use and not just when soap and water are unavailable. Alcohol-based hand-rubs have been used extensively for years in Europe. In the United States, their use was limited to situations in which soap and water were unavailable. Alcohol-based hand-rubs have been proven effective, and they may help improve adherence to hand-hygiene protocols in many healthcare settings. The 2002 CDC hand-hygiene guideline states that alcohol-based hand sanitizers can significantly reduce microorganisms on skin. These hand sanitizers are fast acting and cause less skin irritation. Alcohol-based hand sanitizers for use in healthcare settings are available as low-viscosity gels, rinses, and foams [65].

Proper hand hygiene and care are commonly overlooked asepsis areas for healthcare workers (HCWs). With regards to routine, nonsurgical dental or medical procedures, hand hygiene is mandatory before treatment; between patient appointments; after glove removal; before re-gloving after removing gloves that are cut, torn, or punctured; and before leaving treatment areas.

For surgical procedures, a more rigorous procedure is recommended using a scrub technique to clean nails, hands, and forearms with a surgical antiseptic and a soft sterile brush or sponge. The HCWs should lather for 2 to 6 min using multiple scrub and rinse cycles and dry with sterile towels before donning sterile surgical gloves [62,63,65]. 

Although routine handwashing is a fundamental application of aseptic technique, it can also be a frequent source of dermatitis or exudative problems, which can have immunologic or nonspecific irritant causes. HCWs who have exudative lesions or weeping dermatitis should refrain from direct patient contact until the condition is resolved. [66] They can also take steps to return damaged skin to epithelial integrity by ceasing to use antiseptics that remove skin oils and replace them with a non-antiseptic, mechanical cleansing agent, such as liquid soap and water.

Dryness resulting from frequent handwashing can be relieved by hand lotions which can prevent dermatitis resulting from glove use. Petroleum-based lotion formulations can weaken latex gloves and cause increased permeability. Lotions that contain petroleum or other oil emollients may affect the integrity of gloves and should not be used. At the time of product selection, information should be obtained from the manufacturer regarding interaction between gloves and lotions [63,65].

## 6. Personal Protective Equipment

Since SAR-CoV-2 transmission can occur via direct or indirect contact on the ocular, oral or nasal mucosa with infected body fluids, droplet or aerosol. Healthcare providers need to consider these transmission routes and protect themselves with disposable personal protective equipment (PPE) [67]. PPE recommendations include the use of face shields or protective glasses, fully body disposable gowns, respirators or masks, and gloves. Hand sanitization is a must before and during the donning of PPE.

SARS-CoV-2 can be transmitted through ocular mucous membranes via infectious particles contacting the conjunctival epithelium [67]. Thus, during medical and dental procedures, protective eyewear or a face shield should be used during patient treatment and disinfected after every patient.

Respiratory protection provided by masks or higher-level respirators should always be used for patient care procedures. Aerosol-producing procedures require masks with higher filtration such as a particulate respirator with National Institute for Occupational Safety and Health (NIOSH)-certified N95, European Standard Filtering Face Piece 2 (EU FFP2), or an equivalent. In an emergency involving a suspected COVID-19 patient, an even higher level of respiratory protection such as the respirators conforming to European Standard 149 (EN149) may be required [67]. In addition, fit testing and seal checks are needed to ensure respirator effectiveness.

In addition, inpatients and outpatients in the medical or dental facilities should be required always to wear face masks. 

## 7. Removal of Contaminated Air

Contaminated air in treatment operatories can be removed with use of high-volume evacuators (HVE) or, more expensively, with use of high efficiency particulate air (HEPA) filters. HEPA filtration devices can remove 99.97% of the particles measuring 0.3 μm in diameter. HVE can also remove 90% of contaminated air from the operating site if held 6–15 mm from the aerosol-producing area [68]. HVE remove large volumes of air in a short amount of time. Some hospital high vacuums do not remove large volumes of air and may not be consider HVE. The high vacuum used in dentistry has an opening of at least 8 mm and can remove large volumes of air up to 100 cubic feet of air per minute [39]. However, an assistant or a modification of the HVE may be required to maintain an effective evacuating distance.

The management of potentially contaminate aerosols are best controlled by evacuation procedures using suction devices in the hospital examination rooms. In addition, opening doors and windows may be an effective management for aerosol transmission regardless of the humidity [69]. 

In the ICU, the respiratory equipment for patients must be protected with a high efficiency filter (e.g., BS EN 13328-1) [70], which is disposed after each use. A closed suctioning system is recommended.

In operating rooms, laminar flow ventilation should remain on during all surgical procedures. For patients with suspected or confirmed COVID-19, aerosol-generating procedures should be conducted when essential.

Dental procedures utilizing an assistant and HVE can more effectively reduce the number of colony forming units (CFU) airborne during dental procedures [20]. Incorporation of ultraviolet germicidal irradiation incorporated in HEPA filtration units is also a choice to disinfect environmental air; but can also be expensive.

## 8. Environmental Surface Disinfection

SARS Cov-2 virus survives on inanimate surfaces, thus demonstrating the aerosol viability of the COVID-19 virus. The issue of inanimate surfaces including plastic and stainless steel are especially significant in medical and dental environments, but also applicable for everyday living. Using culture techniques, SARS CoV-2 has been reported to survive on plastic surfaces up to 72 h, and reported to survive on stainless steel surfaces for up to 48 h. Therefore, preventive strategies for surface disinfection for SARS CoV-2 is significant for the reduction of the risk of virus transmission [71].

During patient encounters, fluids having infectious pathogens could contaminate surrounding surfaces. The decontamination of these operatory treatment surfaces between patient appointments constitutes an important component of an effective IC program [72,73]. The routine use of chemical disinfectants or disposable barriers or both is warranted in certain instances because it is not possible, or necessary, to sterilize all contaminated items or surfaces.

Many commercial products are available for use as surface disinfectants. Some of these are capable of surface cleaning and disinfection, some only disinfect, and some have an unpleasant odor, whereas others stain or bleach surfaces. It is essential that before a surface disinfectant is purchased for routine use, medical and dental professionals obtain as much information as possible. This will allow subsequent decisions to be based on appropriate efficacy criteria and reduce the potential for product misuse. Certain products can be used for both surface cleaning and effective environmental surface disinfection, whereas others are useful disinfectants but require initial surface cleaning with another water-based solution.

Social distancing, vaccinations and boosters, air filtration, efficient environmental sanitization, instrument sterilization, and informed use of PPE can significantly reduce the transmission risks. With a better understanding of how SARS-CoV-2 can survive and spread, healthcare providers can better protect themselves and their patients.

## 9. Prevention of Airborne Transmission of Viruses

Single rooms of isolation areas should be appointed for patients with possible or confirmed COVID-19. Single rooms should also be reserved for aerosol-generating procedures. In situations where single rooms are unavailable, suspected COVID-19 patients may be cohorted in a well-ventilated room, and face masks offered to patients. Physical separation to reduce airborne transmission in the hospital setting may include spatial barriers using glass or plastic partitions to manage patients in triage areas and hospital reception areas, as well as curtains around each bed in inpatient wards.

Particle size and airborne infectivity is primarily responsible for COVID transmission between individuals. However, difficulty in controlling environmental variables has been proven to make the interpretation of risk factors difficult. Sampling difficulties such as humidity, wind, temperature, and other environmental issues make interpretation of data inconclusive. In addition, smaller droplets evaporate faster to expose SARS-CoV-2 virions in the environment; this may reduce the viability of the virus. 

Other reasons for reduced emphasis on airborne transmission could be that the smaller droplets have a smaller number of viruses as compared to larger droplets. Furthermore, these small droplets containing SARS-CoV-2 might also physically combine with or attach to pre-existing particulate matter so that their behavior and fate may be governed by the particulate matter composition. Thus, the measurement of their infectivity and viability is highly uncertain due to a lack of a robust sampling system to separately collect virions in the atmosphere [74,75,76].

## 10. Vaccination

The effectiveness of vaccines to control and greatly limit the spread of many human infectious diseases, such as polio, diphtheria, measles, rubella, and tetanus, is regarded as one of the major public health achievements of the 20th century. Vaccinations and boosters can reduce transmissibility of SARS-CoV-2 infection between the vaccinated and non-vaccinated population [77]. This is one strategy for healthcare professionals to reduce their risk of COVID-19 infection. 

Data currently show that the mRNA vaccines against SARS-CoV-2 may not have the longevity and widespread protection against Omicron variants as previously expected. Currently, the following subvariant XBB.1.5, BQ.1.1 and BQ.1 are circulating. Updated bivalent COVID-19 vaccine and booster may protect against the omicron variants and the other COVID variants. The booster dose rate may need to increase to protect against severe complications, hospitalization and death. Therefore, further longitudinal clinical observations are necessary to determine the long-term efficacy of SARS-CoV-2 vaccines.

Thus, the vaccine could reduce COVID-19 symptoms in those vaccinated. The vaccine might reduce but not eliminate the risk of morbidity or mortality of COVID-19. 

## 11. Guidance for Health Care Settings

Current guidelines by the centers for disease control indicate that asymptomatic health care workers who have had higher exposure risks do not require previous work restrictions, despite previous vaccinations or COVID infections [78]. Asymptomatic health care workers must not have active symptoms or test positive for SARS-CoV-2.

Testing frequency may need to be increased to identify variants and potential non-identified variants; this includes variants with shortened incubation periods. In addition, the risk for false negative antigen tests in patients without symptoms should be addressed. Screening of asymptomatic healthcare providers is at the discretion of the director of the health care facility [78]. 

## 12. Conclusions

Transmission of SARS-CoV-2 occurs via aerosols, droplets, and direct contact. The airborne nature of transmission of SARS-CoV-2 can put healthcare providers at a higher risk of infection. Aerosol generation in the healthcare facility can increase the risk of airborne transmission. Aerosols may have viable viruses including SARS-CoV-2. Prolonged suspension of virus-containing aerosols in the environment can carry a risk of airborne transmission for an extended duration. Aerosolized viruses will also be deposited over a larger area. Careful screening of patients via a telephone survey can help screen out COVID-19 patients before the office appointment. Rapid SARS-CoV-2 diagnostic tests can also be used to identify symptomatic or asymptomatic patients.

Social distancing, vaccinations and boosters, air filtration, efficient environmental sanitization, instrument sterilization, and informed use of PPE can significantly reduce the transmission risks. Contaminated air can be removed by HVE and HEPA filters. Tracking infection rates among healthcare providers and correlating it to PPE usage can identify areas where PPE may be inadequate and need reinforcement. To help guide appropriate infection control strategies, further air sampling studies are needed to understand the transmission risk of aerosol-producing procedures. With a better understanding of how SARS-CoV-2 can survive and spread, healthcare providers can better protect themselves and their patients.

## Figures and Tables

**Table 1:** Medical procedures generating aerosols.

Aerosol Generation	Procedures
Head and neck	High-speed cutting, tracheal intubation and extubation, manual ventilation, suctioning of upper ear nose and throat airway, tracheotomy/tracheostomy procedures, sputum induction using nebulized saline
Pulmonary	Bronchoscopy, respiratory tract procedures, high-frequency oscillatory ventilation, high-flow nasal oxygen
Gastrointestinal	Endoscopy, colonoscopy
Orthopedics	High-speed cutting
Post-mortem	High-speed cutting

**Table 2:** Dental devices and procedures that produce airborne contamination.

Dental Devices	Procedures	Airborne Contamination
Ultrasonic and piezoelectric scalers	Oral prophylaxis Scaling and root planning	Greatest source of aerosolized contamination. HVE reduced aerosolized bacteria by almost 99%
Air polishing	Oral prophylaxis Stain removal	Aerosolized bacterial counts were almost as high as ultrasonic scalers. Suction devices reduced aerosolized bacteria by greater than 95%
Air-water syringe	Washing away debris. Air-drying tooth preparation	Bacterial counts aerosolized is almost as high as ultrasonic scalers. HVE reduced aerosolized bacteria by almost 99%
Air turbine handpiece	Tooth preparation	Can be minimal if rubber dam is used
Air abrasion	Tooth preparation	Extensive airborne contamination with abrasive particles, contamination with bacteria is unknown

## Data Availability

Not applicable.
